# Supplementary material for: Effects of urban green spaces on human perceived health improvements: Provision of green spaces is not enough but how people use them matters
Source: PLoS One. 2020 Sep 23;15(9):e0239314. doi: 10.1371/journal.pone.0239314 (PMC7510974; doi:10.1371/journal.pone.0239314)
Supplement: S1 File — (DOC) [file pone.0239314.s001.doc]

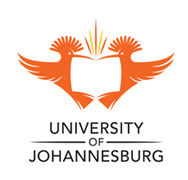


**S1 File**

| Date: | Community: |
| --- | --- |
| Respondent number: |  |

1. **Demographic information**

**1 Gender**

Male
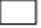
 Female
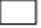


**2 What is your age group range?**

18-29 years

30-40 years

41-50 years

51-60 years

61-64

1. years and older
2. **Level of education?**

Primary level

Secondary

Tertiary

1. **What is your Status?**

Self-employed Employed full-time Employed part-time

Unemployed Retired

1. **Where do you live?**

Low residential area High residential area

1. **Semi-structured approach was guided by the following questions:**

**6. Measuring Urban Green Space Exposure**

- How often do you visit urban green spaces?

| Daily | Weekly | Monthly | On occasion |
| --- | --- | --- | --- |

- How long on average lasts your visit?

| An hour or less | 2-3hours | 4 hours or more |
| --- | --- | --- |

- What are the different activities that you do during each visit?

| Physical exercises | Playing games | Socializing | Meditation | Only passive contact (e.g. just pass by) |
| --- | --- | --- | --- | --- |

**7. Measuring Urban Green Space Provision**

- How many urban green spaces are there in your area?

|  |  |  |
| --- | --- | --- |

- What is the size (km2) of each UGS (Question to City Council)?
- How far is the closest Urban Green space to you?

| 300-500m | 500-750m | > 750 m |
| --- | --- | --- |

- Is access to urban green space in your area free of charge? Yes
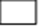
 No **
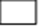
**
- Are there extra facilities provided within urban green spaces in your area? E.g. benches, running tracks, swings.

Yes
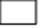
 No
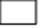


- Rank the quality of green spaces in your area

| zero Quality | Poor quality | Average quality | Good quality | High quality |
| --- | --- | --- | --- | --- |

**8. Moderators**

Cite any or all factors or reasons why you will not visit any Urban Green Space in Bulawayo?

**9. Mediators**

What are the reasons or factors that drive your motivation to visit any green spaces?

**10. Health Responses**

- Do you feel that green spaces impact on your health? Yes
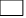
 No
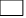

- Do you link any Health improvement to your visit to Urban Green space?

Yes
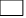
 No
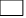


- If Yes, what are the health conditions that you believe got improved by visiting urban green space?

Asthma
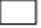
 diabetes
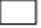
 heart disease
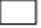
 hypertension
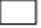
 obesity
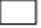
 respiratory problems
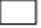
 depression
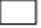
 anxiety
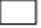
 none of the above

- How much do you think the greenness of the area you reside in contribute to the quality of human health?

| Not much | Significantly | Very much significantly |
| --- | --- | --- |
