# Supplementary material for: Effects of urban green spaces on human perceived health improvements: Provision of green spaces is not enough but how people use them matters
Source: PLoS One. 2020 Sep 23;15(9):e0239314. doi: 10.1371/journal.pone.0239314 (PMC7510974; doi:10.1371/journal.pone.0239314)
Supplement: S2 File — (DOC) [file pone.0239314.s002.doc]

S2 File. R script used for data analysis

#### Testing Zhang et al. 2017 metamodel

# By Dr Kowiyou Yessoufou, University of Johannesburg, South Africa

rm(list=ls(all=TRUE))

library(piecewiseSEM)

library(MASS)

### Accessing the dataset to be analysed on my laptop

dat_ugs <- read.table("C:\\Users\\kowiyouy\\Desktop\\UJ\\MSc\\2018\\Mercy\\Final\\data\\data_ugs.txt",header=TRUE)

attach(dat_ugs)

names(dat_ugs)

###create model1 by converting Figure 1 into an SEM

Sem_Zhang1=list(

glm(perception_in_relation_to_health~quantity*education_level,family=binomial,data=dat_ugs),

glm(frequency_in_a_month~perception_in_relation_to_health+quantity,data=dat_ugs),

glm(as.numeric(mediator_motivation)~frequency_in_a_month,data=dat_ugs),

glm(health_response~frequency_in_a_month*education_level+as.numeric(mediator_motivation)+education_level*quantity,family=binomial, data=dat_ugs)

)

sem.fit(sem_Zhang1, dat_ugs, .progressBar = FALSE)

(coef.table = sem.coefs(sem_Zhang1, dat_ugs))

## in model 1, replace frequency with duration to create model 2

sem_Zhang2=list(

glm(perception_in_relation_to_health~quantity*education_level,family=binomial,data=dat_ugs),

glm(duration_hour~perception_in_relation_to_health+quantity,data=dat_ugs),

glm(as.numeric(mediator_motivation)~duration_hour,data=dat_ugs),

glm(health_response~duration_hour*education_level+as.numeric(mediator_motivation),family=binomial, data=dat_ugs)

)

sem.fit(sem_Zhang2, dat_ugs, .progressBar = FALSE)

(coef.table = sem.coefs(sem_Zhang2, dat_ugs))

## replace duration with intensity to create model 3

sem_Zhang3=list(

lm(perception_in_relation_to_health~quantity*education_level,family="binomial",data=dat_ugs),

glm(intensity~perception_in_relation_to_health+quantity,family="binomial",data=dat_ugs),

glm(as.numeric(mediator_motivation)~intensity,data=dat_ugs),

glm(health_response~intensity*education_level+as.numeric(mediator_motivation),family=binomial, data=dat_ugs)

)

sem.fit(sem_Zhang3, dat_ugs, .progressBar = FALSE)

(coef.table = sem.coefs(sem_Zhang3, dat_ugs))

## Now in Sem_zhang1, lets replace quantity with quality to create model 4

sem_Zhang1.1=list(

glm(perception_in_relation_to_health~quality*education_level,family="binomial",data=dat_ugs),

glm(frequency_in_a_month~perception_in_relation_to_health+quality,data=dat_ugs),

glm(as.numeric(mediator_motivation)~frequency_in_a_month+quality,data=dat_ugs),

glm(health_response~frequency_in_a_month*education_level+as.numeric(mediator_motivation)+education_level*quality,family=binomial, data=dat_ugs)

)

sem.fit(sem_Zhang1.1, dat_ugs, .progressBar = FALSE)

(coef.table = sem.coefs(sem_Zhang1.1, dat_ugs))

## Now in Sem_zhang1.1, lets replace frequency with duration => model 5

sem_Zhang2.1=list(

glm(perception_in_relation_to_health~quality*education_level,family="binomial",data=dat_ugs),

glm(duration_hour~perception_in_relation_to_health+quality,data=dat_ugs),

glm(as.numeric(mediator_motivation)~duration_hour+quality,data=dat_ugs),

glm(health_response~duration_hour*education_level+as.numeric(mediator_motivation)+education_level*quality,family=binomial, data=dat_ugs)

)

sem.fit(sem_Zhang2.1, dat_ugs, .progressBar = FALSE)

(coef.table = sem.coefs(sem_Zhang2.1, dat_ugs))

## Now in Sem_zhang2.1, lets replace duration with intensity => model 6

sem_Zhang3.1=list(

glm(perception_in_relation_to_health~quality*education_level,family="binomial",data=dat_ugs),

glm(intensity~perception_in_relation_to_health+quality,family="binomial",data=dat_ugs),

glm(as.numeric(mediator_motivation)~intensity+quality,data=dat_ugs),

glm(health_response~intensity*education_level+as.numeric(mediator_motivation)+education_level*quality,family=binomial, data=dat_ugs)

)

sem.fit(sem_Zhang3.1, dat_ugs, .progressBar = FALSE)

(coef.table = sem.coefs(sem_Zhang3.1, dat_ugs))

# In sem_zhang1, replace quantity with accessibility = free/charged => model 7

sem_Zhang1.1.1=list(

glm(perception_in_relation_to_health~accessibility_charge*education_level,family=binomial,data=dat_ugs),

glm(frequency_in_a_month~perception_in_relation_to_health+accessibility_charge,data=dat_ugs),

glm(as.numeric(mediator_motivation)~frequency_in_a_month,data=dat_ugs),

glm(health_response~frequency_in_a_month*education_level+as.numeric(mediator_motivation)+education_level*accessibility_charge,family="binomial", data=dat_ugs)

)

sem.fit(sem_Zhang1.1.1, dat_ugs, .progressBar = FALSE)

(coef.table = sem.coefs(sem_Zhang1.1.1, dat_ugs))

# In sem_zhang1.1.1, replace frequency with duration=> model 8

sem_Zhang2.1.1=list(

glm(perception_in_relation_to_health~accessibility_charge*education_level,family=binomial,data=dat_ugs),

glm(duration_hour~perception_in_relation_to_health+accessibility_charge,data=dat_ugs),

glm(as.numeric(mediator_motivation)~duration_hour,data=dat_ugs),

glm(health_response~duration_hour*education_level+as.numeric(mediator_motivation)+education_level*accessibility_charge,family="binomial", data=dat_ugs)

)

sem.fit(sem_Zhang2.1.1, dat_ugs, .progressBar = FALSE)

(coef.table = sem.coefs(sem_Zhang2.1.1, dat_ugs))

# In sem_zhang2.1.1, replace duration with intensity => model 9

sem_Zhang3.1.1=list(

glm(perception_in_relation_to_health~accessibility_charge*education_level,family=binomial,data=dat_ugs),

glm(intensity~perception_in_relation_to_health+accessibility_charge,family="binomial",data=dat_ugs),

glm(as.numeric(mediator_motivation)~intensity,data=dat_ugs),

glm(health_response~intensity*education_level+as.numeric(mediator_motivation)+education_level*accessibility_charge,family="binomial", data=dat_ugs)

)

sem.fit(sem_Zhang3.1.1, dat_ugs, .progressBar = FALSE)

(coef.table = sem.coefs(sem_Zhang3.1.1, dat_ugs))

# In sem_zhang1.1.1, replace accessibility = free/charged with accessibility=distance => model 10

sem_Zhang1.1.1.1=list(

glm(perception_in_relation_to_health~accessibility_distance_m*education_level,family=binomial,data=dat_ugs),

glm(frequency_in_a_month~perception_in_relation_to_health+accessibility_distance_m,data=dat_ugs),

glm(as.numeric(mediator_motivation)~frequency_in_a_month,data=dat_ugs),

glm(health_response~frequency_in_a_month*education_level+as.numeric(mediator_motivation)+education_level*accessibility_distance_m,family="binomial", data=dat_ugs)

)

sem.fit(sem_Zhang1.1.1.1, dat_ugs, .progressBar = FALSE)

(coef.table = sem.coefs(sem_Zhang1.1.1.1, dat_ugs))

# In sem_zhang1.1.1.1, replace frequency with duration => model 11

sem_Zhang2.1.1.1=list(

glm(perception_in_relation_to_health~accessibility_distance_m*education_level,family=binomial,data=dat_ugs),

glm(duration_hour~perception_in_relation_to_health+accessibility_distance_m,data=dat_ugs),

glm(as.numeric(mediator_motivation)~duration_hour,data=dat_ugs),

glm(health_response~duration_hour*education_level+as.numeric(mediator_motivation)+education_level*accessibility_distance_m,family="binomial", data=dat_ugs)

)

sem.fit(sem_Zhang2.1.1.1, dat_ugs, .progressBar = FALSE)

(coef.table = sem.coefs(sem_Zhang2.1.1.1, dat_ugs))

# Finally, in sem_zhang2.1.1.1, replace duration with intensity => model 12

sem_Zhang3.1.1.1=list(

glm(perception_in_relation_to_health~accessibility_distance_m*education_level,family="binomial",data=dat_ugs),

glm(intensity~perception_in_relation_to_health+accessibility_distance_m,family="binomial",data=dat_ugs),

glm(as.numeric(mediator_motivation)~intensity,data=dat_ugs),

glm(health_response~intensity*education_level+as.numeric(mediator_motivation)+education_level*accessibility_distance_m,family="binomial", data=dat_ugs)

)

sem.fit(sem_Zhang3.1.1.1, dat_ugs, .progressBar = FALSE)

(coef.table = sem.coefs(sem_Zhang3.1.1.1, dat_ugs))
