# Supplementary material for: Effects of urban green spaces on human perceived health improvements: Provision of green spaces is not enough but how people use them matters
Source: PLoS One. 2020 Sep 23;15(9):e0239314. doi: 10.1371/journal.pone.0239314 (PMC7510974; doi:10.1371/journal.pone.0239314)
Supplement: S1 Table — (DOC) [file pone.0239314.s003.doc]

**S1 Table.** All data collected and analyzed in this study

| **gender** | **age** | **education_level** | **employment_status** | **residential_area** | **frequency_in_a_month** | **duration_hour** | **intensity** | **quantity** | **accessibility_distance_m** | **accessibility_charge** | **extra_facilities** | **quality** | **moderator** | **mediator_motivation** | **health_response** | **perception_in_relation_to_health** | **health_condition** | **ranking_ugs_effect_on_health** |
| --- | --- | --- | --- | --- | --- | --- | --- | --- | --- | --- | --- | --- | --- | --- | --- | --- | --- | --- |
| Male | 18-29 | tertiary | full_time | high | 0.5 | 1 | 0 | 1 | 1000 | free | no | 1 | not_maintained | neutral | 1 | 0 | NA | not_significant |
| Female | 18-29 | tertiary | full_time | high | 0.5 | 1 | 0 | 1 | 1000 | free | no | 0 | not_maintained | neutral | 1 | 1 | NA | not_significant |
| Female | 18-29 | secondary | unemployed | high | 0.5 | 4 | 0 | 0 | NA | free | yes | 2 | distance | convenience | 1 | 1 | depression | significant |
| Female | 18-29 | secondary | self_employed | high | 30-40 | 1 | 0 | 1 | 700 | free | no | 0 | not_maintained | neutral | 1 | 1 | depression | not_significant |
| Male | 18-29 | tertiary | self_employed | high | 0.5 | 4 | 0 | 0 | NA | charged | yes | 3 | distance_cost | beauty | 1 | 1 | depression | not_significant |
| Male | 18-29 | secondary | unemployed | high | 0.5 | 2 | 0 | 0 | NA | charged | yes | 2 | distance | convenience | 1 | 1 | anxiety | significant |
| Male | 30-40-40 | secondary | unemployed | high | 0.5 | 2 | 0 | 0 | 1000 | free | no | 2 | distance_cost | convenience | 1 | 1 | hypertension | significant |
| Male | 18-29 | secondary | part_time | high | 1 | 4 | 1 | 0 | 1000 | free | yes | 3 | cost | space | 1 | 1 | obesity | significant |
| Female | 30-40-40 | secondary | self_employed | high | 0.5 | 1 | 0 | 1 | 1000 | free | no | 2 | not_maintained | calm | 1 | 0 | depression | significant |
| Female | 50 | secondary | unemployed | high | 4 | 1 | 1 | 1 | 1000 | free | no | 2 | distance | calm | 1 | 1 | depression | not_significant |
| Female | 51 | primary | self_employed | high | 0.5 | 2 | 0 | 0 | 1000 | free | no | 1 | security | space_cool | 1 | 1 | hypertension | very_significant |
| Female | 18-29 | tertiary | part_time | high | 4 | 1 | 1 | 0 | 1000 | free | no | 2 | distance_cost | space | 1 | 1 | obesity_hypertension | significant |
| Male | 30-40 | secondary | full_time | high | 0.5 | 2 | 0 | 0 | 1000 | charged | yes | 3 | distance | convenience | 1 | 1 | anxiety | very_significant |
| Male | 18-29 | secondary | unemployed | high | 4 | 4 | 1 | 0 | 1000 | free | no | 2 | security | space | 1 | 1 | depression | not_significant |
| Male | 18-29 | tertiary | full_time | low | 0.5 | 2 | 0 | 3 | 500 | free | yes | 2 | weather_secluded | beauty | 1 | 1 | anxiety | not_significant |
| Male | 18-29 | secondary | part_time | high | 4 | 4 | 1 | 0 | 1000 | free | yes | 2 | distance | space | 1 | 1 | obesity | significant |
| Female | 30-40 | tertiary | full_time | high | 0.5 | 2 | 0 | 0 | 1000 | free | no | 1 | not_maintained | convenience | 0 | 1 | anxiety | significant |
| Female | 30-40 | tertiary | full_time | high | 0.5 | 4 | 0 | 0 | 1000 | free | no | 1 | distance_cost | convenience | 1 | 1 | depression | significant |
| Female | 18-29 | tertiary | part_time | high | 1 | 4 | 1 | 0 | 1000 | free | no | 1 | distance | calm | 1 | 1 | depression | significant |
| Female | 51 | secondary | retired | high | 0.5 | 1 | 0 | 1 | 500 | free | yes | 0 | not_maintained | neutral | 1 | 1 | NA | not_significant |
| Male | 18-29 | secondary | full_time | high | 0.5 | 1 | 0 | 1 | 1000 | free | yes | 1 | not_maintained | space | 1 | 1 | NA | not_significant |
| Male | 30-40 | tertiary | full_time | low | 0.5 | 1 | 0 | 2 | 1000 | free | no | 1 | not_maintained | outdooor | 1 | 1 | NA | not_significant |
| Male | 18-29 | tertiary | full_time | low | 30 | 1 | 1 | 2 | 500 | restricted | yes | 2 | not_maintained | quiet | 1 | 1 | obesity | not_significant |
| Female | 18-29 | tertiary | part_time | low | 30 | 1 | 1 | 2 | 500 | free | yes | 1 | not_maintained | relaxation | 1 | 1 | hypertension | not_significant |
| Female | 18-29 | secondary | self_employed | low | 4 | 1 | 0 | 1 | 500 | restricted | yes | 1 | not_maintained | convenience | 1 | 0 | depression | significant |
| Male | 18-29 | secondary | unemployed | high | 4 | 2 | 1 | 1 | 1000 | free | yes | 2 | not_maintained | beauty | 1 | 1 | depression | significant |
| Male | 18-29 | secondary | part_time | high | 4 | 2 | 1 | 1 | 500 | restricted | yes | 3 | distance_restricted | beauty | 1 | 1 | depression | not_significant |
| Male | 18-29 | secondary | self_employed | high | 0.5 | 2 | 0 | 1 | 500 | free | no | 0 | not_maintained | calm | 1 | 1 | depression | significant |
| Male | 41-50 | primary | self_employed | low | 30 | 1 | 1 | 2 | 500 | free | yes | 1 | weather | open_space | 1 | 0 | anxiety_depression | significant |
| Female | 18-29 | secondary | unemployed | low | 30 | 1 | 1 | 1 | 500 | free | yes | 1 | absence_facility | open_space | 1 | 1 | NA | significant |
| Female | 30-40 | tertiary | full_time | low | 30 | 1 | 0 | 2 | 500 | free | yes | 1 | not_maintained | open_space | 1 | 1 | hypertension | significant |
| Female | 30-40 | tertiary | full_time | high | 30 | 1 | 0 | 1 | 500 | restricted | no | 3 | access_limited | beauty | 0 | 1 | NA | not_significant |
| Female | 18-29 | tertiary | full_time | low | 4 | 1 | 1 | 3 | 500 | free | yes | 1 | distance | open_space | 1 | 1 | obesity | not_significant |
| Female | 30-40 | secondary | unemployed | high | 0.5 | 2 | 1 | 1 | 1000 | charged | yes | 3 | distance | therapeutic | 1 | 1 | depression | significant |
| Male | 30-40 | tertiary | part_time | low | 0.5 | 1 | 0 | 0 | 1000 | free | yes | 2 | not_maintained | calm | 1 | 1 | depression_hypertension | significant |
| Male | 18-29 | secondary | part_time | high | 30 | 1 | 0 | 1 | 1000 | free | no | 1 | not_maintained | outdooor | 0 | 1 | NA | not_significant |
| Male | 18-29 | secondary | unemployed | high | 4 | 1 | 0 | 2 | 500 | restricted | yes | 2 | not_maintained | space | 1 | 1 | depression | significant |
| Male | 30-40 | secondary | unemployed | high | 0.5 | 1 | 1 | 1 | 1000 | free | no | 1 | not_maintained | open_space | 1 | 1 | obesity | significant |
| Female | 18-29 | secondary | full_time | high | 0.5 | 2 | 0 | 2 | 1000 | free | yes | 3 | absence_facility | space | 0 | 1 | NA | not_significant |
| Female | 18-29 | tertiary | part_time | low | 0.5 | 4 | 0 | 2 | 1000 | free | yes | 3 | distance | greenness | 1 | 1 | depressio | significant |
| Female | 30-40 | tertiary | full_time | high | 4 | 1 | 0 | 0 | 700 | free | no | 0 | security_not_maintained | cool | 0 | 0 | NA | not_significant |
| Male | 18-29 | tertiary | unemployed | low | 0.5 | 4 | 0 | 2 | 700 | charged | yes | 3 | cost | beauty | 1 | 1 | depression | not_significant |
| Male | 30-40 | secondary | Self-employed | high | 0.5 | 2 | 1 | 0 | 1000 | free | no | 0 | absence_facility | space | 1 | 1 | depression_obesity | significant |
| Male | 18-29 | secondary | part_time | low | 4 | 1 | 1 | 3 | 500 | free | yes | 3 | security_secluded | cool_green | 1 | 1 | obesity_hypertension | significant |
| Female | 41-50 | secondary | unemployed | high | 4 | 1 | 1 | 1 | 1000 | free | no | 0 | secluded | space_cool | 1 | 1 | NA | significant |
| Male | 18-29 | tertiary | Self-employed | high | 0.5 | 1 | 0 | 1 | 700 | free | no | 0 | not_maintained | green_open | 0 | 1 | NA | not_significant |
| Female | 18-29 | tertiary | full_time | high | 30 | 1 | 0 | 2 | 1000 | free | no | 1 | security_not_maintained | calm | 0 | 0 | NA | not_significant |
| Female | 18-29 | secondary | unemployed | high | 0.5 | 4 | 0 | 1 | 1000 | free | yes | 3 | distance_security | beauty_green | 0 | 0 | NA | not_significant |
| Female | 18-29 | tertiary | full_time | high | 0.5 | 1 | 0 | 1 | 700 | free | no | 1 | not_maintained | calm | 1 | 0 | NA | significant |
| male | 18-29 | tertiary | part_time | low | 1 | 4 | 0 | 2 | 500 | charged | yes | 3 | cost_unavailability | cool_beauty | 0 | 1 | NA | not_significant |
| Male | 30-40 | tertiary | unemployed | cbd | 0.5 | 1 | 0 | 2 | 1000 | free | yes | 2 | not_maintained | green_space | 0 | 1 | NA | not_significant |
| Female | 30-40 | secondary | Self-employed | high | 0.5 | 1 | 0 | 2 | 700 | free | yes | 1 | weather_unvailability | open_space | 0 | 1 | NA | not_significant |
| Male | 30-40 | tertiary | full_time | low | 4 | 1 | 0 | 2 | 500 | free | no | 3 | isolation_bees | cool_calm | 1 | 1 | NA | significant |
| Female | 30-40 | tertiary | unemployed | high | 0.5 | 2 | 0 | 0 | 1000 | charged | yes | 1 | unavailability | quiet_space | 1 | 1 | depression_anxiety | significant |
| female | 18-29 | secondary | unemployed | high | 30 | 1 | 0 | 2 | 700 | free | no | 1 | security_not_maintained | greeness | 0 | 0 | NA | not_significant |
| Female | 30-40 | secondary | self_employed | high | 30 | 1 | 0 | 1 | 500 | free | no | 0 | not_maintained | open_space | 0 | 1 | NA | not_significant |
| Male | 18-29 | tertiary | unemployed | high | 30 | 1 | 0 | 1 | 700 | free | no | 1 | not_maintained | greeness | 0 | 1 | NA | not_significant |
| Male | 18-29 | tertiary | unemployed | high | 4 | 1 | 0 | 2 | 500 | free | no | 1 | not_maintained | calm_open | 0 | 1 | NA | not_significant |
| Female | 18-29 | secondary | self_employed | high | 30 | 1 | 0 | 1 | 700 | free | no | 0 | not_maintained | space | 0 | 0 | NA | not_significant |
| Male | 18-29 | secondary | unemployed | high | 4 | 2 | 1 | 2 | 1000 | free | no | 1 | distance | space | 1 | 1 | obesity_depression | significant |
| Male | 30-40 | tertiary | full_time | high | 30 | 1 | 0 | 1 | 500 | free | no | 1 | unavailability | open_space | 0 | 1 | NA | not_significant |
| Male | 18-29 | secondary | unemployed | high | 30 | 2 | 0 | 2 | 500 | free | no,vandalised | 0 | weather | outdoor_calm | 1 | 1 | depression | not_significant |
| Female | 18-29 | tertiary | unemployed | high | 30 | 1 | 1 | 1 | 500 | free | no | 1 | security_not_maintained | open_space | 1 | 1 | obesity | significant |
| Female | 41-50 | secondary | self_employed | high | 4 | 1 | 0 | 1 | 500 | free | no | 1 | security_secluded | greeness | 0 | 1 | NA | not_significant |
| Male | 30-40 | tertiary | full_time | high | 0.5 | 4 | 0 | 0 | 1000 | charged | yes | 3 | distance_unavailability | open_space | 0 | 0 | NA | not_significant |
| Male | 30-40 | tertiary | full_time | high | 0.5 | 4 | 0 | 0 | 1000 | charged | yes | 3 | distance_unavailability | outdoor_beauty | 0 | 1 | NA | not_significant |
| Female | 18-29 | tertiary | unemployed | high | 30 | 1 | 0 | 1 | 500 | free | no | 0 | not_maintained | greeness | 0 | 0 | NA | not_significant |
| Female | 30-40 | secondary | self_employed | high | 0.5 | 1 | 0 | 1 | 500 | free | no | 1 | security | open_calm | 0 | 1 | NA | not_significant |
| Female | 18-29 | secondary | full_time | high | 30 | 1 | 0 | 1 | 500 | free | no | 0 | security_beauty | space_calm | 0 | 0 | NA | not_significant |
| Male | 18-29 | tertiary | unemployed | high | 4 | 1 | 0 | 0 | 1000 | free | no | 1 | not_maintained | calm_private | 0 | 1 | NA | not_significant |
| male | 18-29 | tertiary | unemployed | low | 4 | 2 | 1 | 2 | 500 | free | yes | 3 | weather_unvailability | space | 1 | 1 | depression_respiratory | significant |
| Male | 18-29 | tertiary | part_time | low | 4 | 2 | 1 | 2 | 500 | free | yes | 2 | unavailability | space | 1 | 1 | obesity | significant |
| Male | 18-29 | tertiary | unemployed | low | 4 | 2 | 1 | 3 | 500 | free | yes | 3 | unavailability | greeness_open | 1 | 1 | obesity | significant |
| Female | 30-40 | tertiary | full_time | low | 0.5 | 4 | 0 | 1 | 1000 | charged | yes | 3 | unavailability | beauty | 1 | 1 | depression | not_significant |
| Female | 18-29 | tertiary | full_time | low | 0.5 | 4 | 0 | 3 | 500 | charged | yes | 3 | weather | beauty | 1 | 1 | depression | significant |
| Female | 18-29 | tertiary | unemployed | low | 0.5 | 4 | 0 | 2 | 700 | charged | yes | 3 | weather_unvailability | natural | 1 | 1 | anxiety | significant |
| Male | 18-29 | secondary | unemployed | high | 4 | 2 | 1 | 0 | 1000 | free | no | 0 | distance | space | 1 | 1 | obesity_depression | significant |
| Female | 30-40 | tertiary | full_time | high | 30 | 1 | 0 | 1 | 700 | free | no | 1 | security_unavailability | open | 0 | 1 | NA | not_significant |
| Male | 18-29 | secondary | full_time | high | 30 | 1 | 0 | 1 | 500 | free | no | 2 | not_maintained | neutral | 0 | 1 | NA | not_significant |
| Male | 41-50 | primary | self_employed | high | 30 | 1 | 0 | 1 | 1000 | free | no | 1 | security | greeness | 0 | 0 | NA | not_significant |
| Male | 18-29 | tertiary | part_time | high | 4 | 1 | 0 | 2 | 1000 | free | yes | 2 | not_maintained | calm | 1 | 1 | depression_anxiety | significant |
| Female | 30-40 | secondary | full_time | low | 4 | 1 | 0 | 2 | 500 | free | no | 2 | unavailability_secluded | calm | 0 | 1 | NA | not_significant |
| Female | 18-29 | tertiary | unemployed | cbd | 0.5 | 4 | 0 | 3 | 1000 | free | yes | 3 | security | natural | 0 | 1 | NA | not_significant |
| Male | 18-29 | secondary | full_time | low | 0.5 | 2 | 0 | 3 | 700 | charged | yes | 3 | distance_unavailability | outdoor | 0 | 1 | NA | not_significant |
| Male | 30-40 | secondary | self_employed | high | 0.5 | 1 | 0 | 1 | 1000 | free | no | 1 | unavailability | open_space | 0 | 0 | NA | not_significant |
| Female | 18-29 | tertiary | unemployed | low | 30 | 1 | 1 | 3 | 500 | free | no | 2 | weather | open_calm | 1 | 0 | obesity | significant |
| Female | 18-29 | tertiary | unemployed | high | 4 | 1 | 0 | 1 | 500 | free | no | 0 | not_maintained | space | 1 | 1 | depression | not_significant |
| Female | 30-40 | secondary | full_time | high | 30 | 1 | 0 | 2 | 700 | free | no | 0 | not_maintained | neutral | 0 | 1 | NA | not_significant |
| Male | 30-40 | secondary | full_time | high | 30 | 1 | 0 | 2 | 500 | free | no | 1 | security_not_maintained | calm | 0 | 0 | NA | not_significant |
| Female | 30-40 | secondary | unemployed | high | 30 | 1 | 0 | 1 | 500 | free | no | 0 | security_not_maintained | NA | 0 | 1 | NA | not_significant |
| Female | 18-29 | secondary | full_time | high | 0.5 | 1 | 0 | 1 | 700 | free | no | 1 | not_maintained | relaxation | 1 | 1 | depression | not_significant |
| Female | 30-40 | secondary | self_employed | high | 0.5 | 4 | 0 | 2 | 500 | charged | yes | 3 | cost_unavailability | cool_beauty | 0 | 1 | NA | not_significant |
| Male | 18-29 | tertiary | unemloyed | high | 0.5 | 1 | 0 | 2 | 1000 | free | yes | 2 | not_maintained | green_space | 0 | 1 | NA | not_significant |
| Male | 41-50 | tertiary | full_time | high | 0.5 | 1 | 0 | 2 | 700 | free | yes | 1 | weather_unvailability | open_space | 0 | 1 | NA | not_significant |
| Female | 18-29 | tertiary | part_time | high | 4 | 1 | 1 | 0 | 1000 | free | no | 2 | cost_distance | space | 1 | 1 | obesity | significant |
| Male | 30-40 | secondary | full_time | high | 0.5 | 2 | 0 | 0 | 1000 | charged | yes | 3 | distance | convenience | 1 | 1 | anxiety | very_significant |
| Female | 18-29 | tertiary | unemployed | high | 30 | 1 | 1 | 1 | 500 | free | no | 1 | security_not_maintained | open_space | 1 | 1 | obesity | significant |
| Female | 41-50 | secondary | self_employed | high | 4 | 1 | 0 | 1 | 500 | free | no | 1 | security_secluded | greeness | 0 | 1 | NA | not_significant |
| Male | 30-40 | tertiary | full_time | high | 0.5 | 4 | 0 | 0 | 1000 | charged | yes | 3 | distance_unavailability | open_space | 0 | 0 | NA | not_significant |
| Female | 30-40 | tertiary | full_time | high | 4 | 1 | 0 | 0 | 700 | free | no | 0 | security_not_maintained | cool | 0 | 0 | NA | not_significant |
| Male | 18-29 | tertiary | unemployed | low | 0.5 | 4 | 0 | 2 | 700 | charged | yes | 3 | cost | beauty | 0 | 1 | NA | not_significant |
