# Supplementary material for: Effects of urban green spaces on human perceived health improvements: Provision of green spaces is not enough but how people use them matters
Source: PLoS One. 2020 Sep 23;15(9):e0239314. doi: 10.1371/journal.pone.0239314 (PMC7510974; doi:10.1371/journal.pone.0239314)
Supplement: S2 Table — See R scripts in S2 File for details of the meta-model. * indicates significant relationships between predictor and response. (DOC) [file pone.0239314.s004.doc]

**S2 Table. Path coefficients of meta-model 1 defined in Figure 2. See R scripts in SI-4 for details of the meta-model. * indicates significant relationships between predictor and response.**

| **response** | **predictor** | **estimate** | **Std.error** | **p.value** |
| --- | --- | --- | --- | --- |
| 1. perception_ in_ relation _to _health | quantity:education_leveltertiary | 32.88983162 | 2.843294e+03 | 0.9908 |
| 1. perception | quantity | -32.34447794 | 2.843294e+03 | 0.9909 |
| 1. perception | quantity:education_levelsecondary | 32.01493791 | 2.843294e+03 | 0.9910 |
| 1. perception | education_leveltertiary | -15.53905425 | 2.272366e+03 | 0.9945 |
| 1. perception | education_levelsecondary | -14.49439288 | 2.272366e+03 | 0.9949 |
| 1. frequency_in_a_month | perception_in_relation_to_healthgood | -7.62896850 | 3.081436e+00 | 0.0150 * |
| 1. frequency_in_a_month | quantity | 2.28614004 | 1.336178e+00 | 0.0903 |
| 1. as.numeric(mediator_motivatio) | frequency_in_a_month | 0.12568750 | 7.777633e-02 | 0.1093 |
| 1. health_response | as.numeric(mediator_motivation) | 0.03759805 | 2.519415e-02 | 0.1356 |
| 1. health_response | frequency_in_a_month | -2.21565833 | 1.992427e+02 | 0.9911 |
| 1. health_response | frequency_in_a_month:education_leveltertiary | 2.19042066 | 1.992427e+02 | 0.9912 |
| 1. health_response | frequency_in_a_month:education_ | 2.15323513 | 1.992427e+02 | 0.9914 |
| 1. health_response | education_levelsecondary: quantity | -34.14295312 | 3.393469e+03 | 0.9920 |
| 1. health_response | quantity | 32.86895054 | 3.393469e+03 | 0.9923 |
| 1. health_response | education_leveltertiary:quantity | -32.44009345 | 3.393469e+03 | 0.9924 |
| 1. health_response | education_leveltertiary | -17.02220580 | 2.441909e+03 | 0.9944 |
| 17.health_response | education_levelsecondary | -14.65677167 | 2.441909e+03 | 0.9952 |
