# Supplementary material for: Effects of urban green spaces on human perceived health improvements: Provision of green spaces is not enough but how people use them matters
Source: PLoS One. 2020 Sep 23;15(9):e0239314. doi: 10.1371/journal.pone.0239314 (PMC7510974; doi:10.1371/journal.pone.0239314)
Supplement: S3 Table — See R scripts in S2 File for details of the meta-model. * indicates significant relationships between predictor and response. (DOC) [file pone.0239314.s005.doc]

**S3 Table.** Path coefficients of meta-model 2 defined in Figure 2. See R scripts in SI-4 for details of the meta-model. * indicates significant relationships between predictor and response.

| **response** | **predictor** | **estimate** | **Std.error** | **p.value** |
| --- | --- | --- | --- | --- |
| 1. perception_ in_ relation _to _health | quantity:education_leveltertiary | 32.88983162 | 2.843294e+03 | 0.9908 |
| 1. perception | quantity | 32.34447794 | 2.843294e+03 | 0.9909 |
| 1. perception | quantity:education_levelsecondary | 32.01493791 | 2.843294e+03 | 0.9910 |
| 1. perception | education_leveltertiary | -15.53905425 | 2.272366e+03 | 0.9945 |
| 1. perception | education_levelsecondary | -14.49439288 | 2.272366e+03 | 0.9949 |
| 1. duration_hour | perception_in_relation_to_healthgood | 0.42495922 | 2.945763e-01 | 0.1523 |
| 1. duration_hour | quantity | -0.18026101 | 1.277347e-01 | 0.1613 |
| 1. as.numeric(mediator_motivation | duration_hour | -1.33060049 | 8.224546e-01 | 0.1089 |
| 1. health_response | as.numeric(mediator_motivation) | 0.02703871 | 2.289535e-02 | 0.2376 |
| 1. health_response | education_leveltertiary | 15.48178095 | 1.455400e+03 | 0.9915 |
| 1. health_response | duration_hour | 15.20104566 | 1.455398e+03 | 0.9917 |
| 1. health_response | duration_hour:education_leveltertiary | -15.11759521 | 1.455398e+03 | 0.9917 |
| 1. health_response | education_levelsecondary | 14.84093182 | 1.455400e+03 1.455398e+03 | 0.9919 |
| 1. health_response | duration_hour:education_levelsecondary | -14.70393816 | 2.843294e+03 | 0.9919 |
