# Supplementary material for: Effects of urban green spaces on human perceived health improvements: Provision of green spaces is not enough but how people use them matters
Source: PLoS One. 2020 Sep 23;15(9):e0239314. doi: 10.1371/journal.pone.0239314 (PMC7510974; doi:10.1371/journal.pone.0239314)
Supplement: S4 Table — See R scripts in S2 File for details of the meta-model. * indicates significant relationships between predictor and response. (DOC) [file pone.0239314.s006.doc]

**S4 Table. Path coefficients of meta-model 3 defined in Figure 2. See R scripts in SI-4 for details of the meta-model. * indicates significant relationships between predictor and response.**

| **response** | **predictor** | **estimate** | **Std.error** | **p.value** |
| --- | --- | --- | --- | --- |
| 1. perception _in _relation _to _health | quantity:education_leveltertiary | 32.88983162 | 2.843294e+03 | 0.9908 |
| 1. perception | quantity | -32.34447794 | 2.843294e+03 | 0.9909 |
| 1. perception | quantity:education_levelsecondary | 32.01493791 | 2.843294e+03 | 0.9910 |
| 1. perception | education_leveltertiary | -15.53905425 | 2.272366e+03 | 0.9945 |
| 1. perception | education_levelsecondary | -14.49439288 | 2.272366e+03 | 0.9949 |
| 1. intensity | perception_in_relation_to_healthgood | 1.31376039 | 7.855603e-01 | 0.0944 |
| 1. intensity | quantity | 0.01889212 | 2.501594e-01 | 0.9398 |
| 1. as.numeric(mediator_motivation) | intensity | 7.86149163 | 2.034683e+00 | 0.0002 *** |
| 1. health_response | as.numeric(mediator_motivation) | -0.02107515 | 2.805162e-02 | 0.4525 |
| 1. health_response | education_levelsecondary | -0.39123104 | 1.476489e+00  1.486178e+00 | 0.7910 |
| 1. health_response | education_leveltertiary | -0.37664327 | 6.522639e+03 | 0.7999 |
| 1. health_response | intensity | 18.53445578 | 6.787604e+03 | 0.9977 |
| 1. health_response | intensity:education_leveltertiary | 0.41729150 | 6.748800e+03 | 1.0000 |
| 14.health_response | intensity:education_levelsecondary | 0.41022005 | 2.843294e+03 | 1.0000 |
