# Supplementary material for: Effects of urban green spaces on human perceived health improvements: Provision of green spaces is not enough but how people use them matters
Source: PLoS One. 2020 Sep 23;15(9):e0239314. doi: 10.1371/journal.pone.0239314 (PMC7510974; doi:10.1371/journal.pone.0239314)
Supplement: S5 Table — See R scripts in S2 File for details of the meta-model. * indicates significant relationships between predictor and response. (DOC) [file pone.0239314.s007.doc]

**S5 Table. Path coefficients of meta-model 4 defined in Figure 2. See R scripts in SI-4 for details of the meta-model. * indicates significant relationships between predictor and response.**

| **response** | **predictor** | **estimate** | **Std.error** | **p.value** |
| --- | --- | --- | --- | --- |
| 1. perception_in_relation_to_health | education_levelsecondary | 2.47593470 | 1.412889e+00 | .0797 |
| 1. perception_in_relation_to_health | education_leveltertiary | 1.93791116 | 1.287403e+00 | 0.1323 |
| 1. perception_in_relation_to_health | quality | 0.58007227 | 3.873225e-01 | 0.1342 |
| 1. perception_in_relation_to_health | quality:education_levelsecondary | -0.28458685 | 5.377334e-01 | 0.5966 |
| 1. frequency_in_a_month | quality | -4.10606984 | 1.096147e+00 | 0.0003 *** |
| 1. frequency_in_a_month | perception_in_relation_to_healthgood | -5.41225685 | 2.967427e+00 | 0.0712 |
| 1. as.numeric(mediator_motivation) | quality | -1.99192611 | 9.608594e-01 | 0.0408 * |
| 1. as.numeric(mediator_motivation) | frequency_in_a_month | 0.06368734 | 8.213825e-02 | 0.4400 |
| 1. health_response | as.numeric(mediator_motivation) | 0.03381942 | 2.437629e-02 | 0.1653 |
| 1. health_response | education_levelsecondary:quality | -0.39300445 | 4.664137e-01 | 0.3994 |
| 1. health_response | quality | 0.20937018 | 2.909437e-01 | 0.4718 |
| 1. health_response | frequency_in_a_month | -0.51218665 | 4.933553e+01 | 0.9917 |
| 1. health_response | education_leveltertiary | -14.90973040 | 1.480065e+03 | 0.9920 |
| 1. health_response | frequency_in_a_month:education_leveltertiary | 0.49646095 | 4.933554e+01 | 0.9920 |
| 1. health_response | education_levelsecondary | -13.73515876 | 1.480066e+03 | 0.9926 |
| 1. health_response | frequency_in_a_month:education_levelsecondary | 0.43467671 | 1.480066e+03 | 0.9930 |
