# Supplementary material for: Effects of urban green spaces on human perceived health improvements: Provision of green spaces is not enough but how people use them matters
Source: PLoS One. 2020 Sep 23;15(9):e0239314. doi: 10.1371/journal.pone.0239314 (PMC7510974; doi:10.1371/journal.pone.0239314)
Supplement: S6 Table — See R scripts in S2 File for details of the meta-model. * indicates significant relationships between predictor and response. (DOC) [file pone.0239314.s008.doc]

**S6 Table. Path coefficients of meta-model 5 defined in Figure 2. See R scripts in SI-4 for details of the meta-model. * indicates significant relationships between predictor and response.**

| **response** | **predictor** | **estimate** | **Std.error** | **p.value** |
| --- | --- | --- | --- | --- |
| 1. perception_in_relation_to_health | education_levelsecondary | 2.47593470 | 1.412889e+00 | 0.0797 |
| 1. perception_in_relation_to_health | education_leveltertiary | 1.93791116 | 1.287403e+00 | 0.1323 |
| 1. perception_in_relation_to_health | quality | 0.58007227 | 3.873225e-01 | 0.1342 |
| 1. perception_in_relation_to_health | quality:education_levelsecondary | -0.28458685 | 5.377334e-01 | 0.5966 |
| 1. duration_hour | quality | 0.62911882 | 9.163935e-02 | 0.0000 *** |
| 1. duration_hour | perception_in_relation_to_healthgood | 0.10474572 | 2.480809e-01 | 0.6738 |
| 1. as.numeric(mediator_motivation) | quality | -2.11980331 | 1.098953e+00 | 0.0567 |
| 1. as.numeric(mediator_motivation) | duration_hour | -0.22458892 | 9.934410e-01 | 0.8216 |
| 1. health response | as.numeric(mediator_motivation) | 0.02742747 | 2.390262e-02 | 0.2512 |
| 1. health response | quality | 0.30400331 | 3.641285e-01 | 0.4038 |
| 1. health response | education_levelsecondary:quality | -0.26368076 | 5.007324e-01 | 0.5985 |
| 1. health response | education_leveltertiary | 15.56639726 | 1.455400e+03 | 0.9915 |
| 1. health response | duration_hour:education_leveltertiary | -15.26580697 | 1.455398e+03 | 0.9916 |
| 1. health response | duration_hour | 15.19579741 | 1.455398e+03 | 0.9917 |
| 1. health response | education_levelsecondary | 15.11914138 | 1.455401e+03 | 0.9917 |
| 1. health response | duration_hour:education_levelsecondary | -14.72102779 | 1.455398e+03 | 0.9919 |
