# Supplementary material for: Effects of urban green spaces on human perceived health improvements: Provision of green spaces is not enough but how people use them matters
Source: PLoS One. 2020 Sep 23;15(9):e0239314. doi: 10.1371/journal.pone.0239314 (PMC7510974; doi:10.1371/journal.pone.0239314)
Supplement: S7 Table — See R scripts in S2 File for details of the meta-model. * indicates significant relationships between predictor and response. (DOC) [file pone.0239314.s009.doc]

**S7 Table. Path coefficients of meta-model 6 defined in Figure 2. See R scripts in SI-4 for details of the meta-model. * indicates significant relationships between predictor and response.**

| **response** | **predictor** | **estimate** | **Std.error** | **p.value** |
| --- | --- | --- | --- | --- |
| 1. perception_in_relation_to_health | education_levelsecondary | 2.47593470 | 1.412889e+00 | 0.0797 |
| 1. perception_in_relation_to_health | education_leveltertiary | 1.93791116 | 1.287403e+00 | 0.1323 |
| 1. perception_in_relation_to_health | quality | 0.58007227 | 3.873225e-01 | 0.1342 |
| 1. perception_in_relation_to_health | quality:education_levelsecondary | -0.28458685 | 5.377334e-01 | 0.5966 |
| 1. intensity | perception_in_relation_to_healthgood | 1.27574435 | 7.913862e-01 | 0.1070 |
| 1. intensity | quality | 0.08703173 | 2.181956e-01 | 0.6900 |
| 1. as.numeric(mediator_motivation) | intensity | 8.22346508 | 1.960471e+00 | 0.0001 *** |
| 1. as.numeric(mediator_motivation) | quality | -2.47725578 | 8.274611e-01 | 0.0035 ** |
| 1. health response | as.numeric(mediator_motivation) | -0.01904312 | 2.947379e-02 | 0.5182 |
| 1. health response | quality | 0.18150755 | 2.964143e-01 | 0.5403 |
| 1. health response | education_levelsecondary:quality | -0.21789779 | 4.515162e-01 | 0.6294 |
| 1. health response | education_leveltertiary | -0.48291909 | 1.496724e+00 | 0.7470 |
| 1. health response | intensity | -0.14697272 | 1.565984e+00 | 0.9252 |
| 1. health response | intensity:education_levelsecondary | 18.53750383 | 6.522639e+03 | 0.9977 |
| 1. health response | duration_hour:education_levelsecondary | 0.40284313 | 6.749431e+03 | 1.0000 |
| 1. health response | intensity:education_leveltertiary | 0.38242302 | 6.786752e+03 | 1.0000 |
