# Supplementary material for: Effects of urban green spaces on human perceived health improvements: Provision of green spaces is not enough but how people use them matters
Source: PLoS One. 2020 Sep 23;15(9):e0239314. doi: 10.1371/journal.pone.0239314 (PMC7510974; doi:10.1371/journal.pone.0239314)
Supplement: S8 Table — See R scripts in SI-4 for details of the meta-model. * indicates significant relationships between predictor and response. (DOC) [file pone.0239314.s010.doc]

**S8 Table. Path coefficients of meta-model 7 defined in Figure 2. See R scripts in SI-4 for details of the meta-model. * indicates significant relationships between predictor and response.**

| **response** | **predictor** | **estimate** | **Std.error** | **p.value** |
| --- | --- | --- | --- | --- |
| 1. perception_in_relation_to_health | education_levelsecondary | 2.18122424 | 1.294262e+00 | 0.0919 |
| 1. perception_in_relation_to_health | education_leveltertiary | 2.18122424 | 1.294262e+00 | 0.0919 |
| 1. perception_in_relation_to_health | accessibility_charge1 | 0.01600034 | 8.866940e-01 | 0.9856 |
| 1. perception_in_relation_to_health | accessibility_charge1:education_levelsecondary | 16.06199109 | 1.615104e+03 | 0.9921 |
| 1. perception_in_relation_to_health | accessibility_chargerestricted:education_levelsecondary | 16.87292130 | 797442e+03 | 0.9952 |
| 1. perception_in_relation_to_health | accessibility_chargerestricted | 16.07799143 | 797442e+03 | 0.9954 |
| 1. frequency_in_a_month | accessibility_charge1 | -9.44669686 | 148077e+00 | 0.0034 ** |
| 1. frequency_in_a_month | perception_in_relation_to_healthgood | -6.61917650 | 2.998025e+00 | 0.0296 * |
| 1. frequency_in_a_month | accessibility_chargerestricted frequency_in_a_month | 3.87878273 | 5.412098e+00 | 0.4753 |
| 1. as.numeric(mediator_motivation) | as.numeric(mediator_motivation) | 0.12568750 | 7.777633e-02 | 0.1093 |
| 1. health response | accessibility_charge1 | 0.03864026 | 2.451875e-02 | 0.1150 |
| 1. health response | perception_in_relation_to_healthgood | -0.34738355 | 7.477419e-01 | 0.6422 |
| 1. health response | accessibility_chargerestricted | 0.28582761 | 1.240310e+00 | 0.8177 |
| 1. health response | education_levelsecondary:accessibility_charge1 | 0.14266171 | 1.588666e+00 | 0.9284 |
| 1. health response | accessibility_chargerestricted | 15.96118201 | 1.342299e+03 | 0.9905 |
| 1. health response | education_levelsecondary:accessibility_chargerestricted frequency_in_a_month | -0.54387881 | 8.134051e+01 | 0.9947 |
| 1. health response | education_leveltertiary | -15.54167371 | 2.440215e+03 | 0.9949 |
| 1. health response | frequency_in_a_month:education_leveltertiary | 0.51780843 | 8.134052e+01 | 0.9949 |
| 1. health response | education_levelsecondary | -15.37336983 | 2.440215e+03 | 0.9950 |
| 1. health response | frequency_in_a_month:education_levelsecondary | 0.47741154 | 8.134052e+01 | 0.9953 |
