# Supplementary material for: Effects of urban green spaces on human perceived health improvements: Provision of green spaces is not enough but how people use them matters
Source: PLoS One. 2020 Sep 23;15(9):e0239314. doi: 10.1371/journal.pone.0239314 (PMC7510974; doi:10.1371/journal.pone.0239314)
Supplement: S9 Table — See R scripts in S2 File for details of the meta-model. * indicates significant relationships between predictor and response. (DOC) [file pone.0239314.s011.doc]

**S9 Table. Path coefficients of meta-model 8 defined in Figure 2. See R scripts in SI-4 for details of the meta-model. * indicates significant relationships between predictor and response.**

| **response** | **predictor** | **estimate** | **Std.error** | **p.value** |
| --- | --- | --- | --- | --- |
| 1. perception_in_relation_to_health | education_levelsecondary | 2.18122424 | .294262e+00 | 0.0919 |
| 1. perception_in_relation_to_health | education_leveltertiary | 2.18122424 | 1.294262e+00 | 0.0919 |
| 1. perception_in_relation_to_health | accessibility_charge1 | 0.01600034 | 8.866940e-01 | 0.9856 |
| 1. perception_in_relation_to_health | accessibility_charge1:education_levelsecondary | 16.06199109 | 1.615104e+03 | 0.9921 |
| 1. perception_in_relation_to_health | accessibility_chargerestricted:education_levelsecondary | 16.87292130 | 797442e+03 | 0.9952 |
| 1. perception_in_relation_to_health | accessibility_chargerestricted | 16.07799143 | 797442e+03 | 0.9954 |
| 1. duration_hour | accessibility_charge1 | 1.75246351 | 2.568789e-01 | 0.0000 *** |
| 1. duration_hour | perception_in_relation_to_healthgood | 0.26703077 | 2.446348e-01 | 0.2777 |
| 1. duration_hour | accessibility_chargerestricted duration_hour | -0.31966337 | 4.416200e-01 | 0.4709 |
| 1. as.numeric(mediator_motivation) | as.numeric(mediator_motivation) | -1.33060049 | 8.224546e-01 | 0.1089 |
| 1. health response | accessibility_charge1 | 0.03253936 | 2.379696e-02 | 0.1715 |
| 1. health response | education_levelsecondary: accessibility_charge1 | -0.81897788 | 1.123247e+00 | 0.4659 |
| 1. health response | accessibility_chargerestricted | 0.99893984 | 1.505915e+00 | 0.5071 |
| 1. health response | education_levelsecondary:accessibility_chargerestricted | -0.26945847 | 1.487328e+00 | 0.8562 |
| 1. health response | education_leveltertiary | 16.87325730 | 1.363783e+03 | 0.9901 |
| 1. health response | duration_hour | 16.22269160 | 2.399546e+03 | 0.9946 |
| 1. health response | duration_hour:education_leveltertiary | 16.12678703 | 2.399545e+03 | 0.9946 |
| 1. health response | duration_hour:education_levelsecondary | -15.83934536 | 399545e+03 | 0.9947 |
| 1. health response | education_levelsecondary | -15.60105116 | 2.399545e+03 | 0.9948 |
| 1. health response | education_leveltertiary | 15.55625816 | 2.399547e+03 | 0.9948 |
