# Supplementary material for: Effects of urban green spaces on human perceived health improvements: Provision of green spaces is not enough but how people use them matters
Source: PLoS One. 2020 Sep 23;15(9):e0239314. doi: 10.1371/journal.pone.0239314 (PMC7510974; doi:10.1371/journal.pone.0239314)
Supplement: S10 Table — See R scripts in S2 File for details of the meta-model. * indicates significant relationships between predictor and response. (DOC) [file pone.0239314.s012.doc]

**S10 Table. Path coefficients of meta-model 9 defined in Figure 2. See R scripts in SI-4 for details of the meta-model. * indicates significant relationships between predictor and response.**

| **response** | **predictor** | **estimate** | **Std.error** | **p.value** |
| --- | --- | --- | --- | --- |
| 1. perception_in_relation_to_health | education_levelsecondary | 2.18122424 2.18122424 | 1.294262e+00 | 0.0919 |
| 1. perception_in_relation_to_health | education_leveltertiary | 0.01600034 | 1.294262e+00 | 0.0919 |
| 1. perception_in_relation_to_health | accessibility_charge1 | 16.06199109 | 8.866940e-01 | 0.9856 |
| 1. perception_in_relation_to_health | accessibility_charge1:education_levelsecondary | 16.87292130 | 1.615104e+03 | 0.9921 |
| 1. perception_in_relation_to_health | accessibility_chargerestricted:education_levelsecondary | 16.07799143 | 797442e+03 | 0.9952 |
| 1. perception_in_relation_to_health | accessibility_chargerestricted | -2.06473831 | 797442e+03 | 0.9954 |
| 1. intensity | accessibility_charge1 | 1.45469850 | 1.064239e+00 | 0.0524 |
| 1. intensity | perception_in_relation_to_healthgood | 0.44325871 | 7.928164e-01 | 0.0665 |
| 1. intensity | accessibility_chargerestricted duration_hour | 7.86149163 | 9.731446e-01 | 0.6488 |
| 1. as.numeric(mediator_motivation) | as.numeric(mediator_motivation) | -0.02433925 | 2.034683e+00 | 0.0002 *** |
| 1. health response | accessibility_charge1 | 0.39119261 | 2.996847e-02 | 0.4167 |
| 1. health response | education_levelsecondary | -0.71854921 | 7.232836e-01 | 0.5886 |
| 1. health response | accessibility_chargerestricted | 0.44511197 | 1.493594e+00 | 0.6305 |
| 1. health response | education_levelsecondary:accessibility_chargerestricted | -0.46873037 | 1.249336e+00 | 0.7216 |
| 1. health response | accessibility_chargerestricted | 36.10121794 | 1.505116e+00 | 0.7555 |
| 1. health response | intensity | -16.75836588 | 5.572547e+03 | 0.9948 |
| 1. health response | intensity:education_leveltertiary | 19.52955964 | 2.602092e+03 | 0.9949 |
| 1. health response | intensity:education_levelsecondary | 14.51086559 | 1.075401e+04 | 0.9986 |
| 1. health response | education_levelsecondary | 0.56299640 | 1.136620e+04 | 0.9990 |
| 1. health response | education_leveltertiary | 2.18122424 | 1.109704e+04 | 1.0000 |
