# Supplementary material for: Effects of urban green spaces on human perceived health improvements: Provision of green spaces is not enough but how people use them matters
Source: PLoS One. 2020 Sep 23;15(9):e0239314. doi: 10.1371/journal.pone.0239314 (PMC7510974; doi:10.1371/journal.pone.0239314)
Supplement: S11 Table — See R scripts in S2 File for details of the meta-model. * indicates significant relationships between predictor and response. (DOC) [file pone.0239314.s013.doc]

**S11 Table. Path coefficients of meta-model 10 defined in Figure 2. See R scripts in SI-4 for details of the meta-model. * indicates significant relationships between predictor and response.**

| **response** | **predictor** | **estimate** | **Std.error** | **p.value** |
| --- | --- | --- | --- | --- |
| 1. perception_in_relation_to_health | education_leveltertiary | 33.37039590 | 2.910796e+03 | 0.9909 |
| 1. perception_in_relation_to_health | education_levelsecondary | 32.39251875 | 2.910796e+03 | 0.9911 |
| 1. perception_in_relation_to_health | accessibility_distance_m:education_leveltertiary | -0.03207553 | 2.910797e+00 | 0.9912 |
| 1. perception_in_relation_to_health | accessibility_distance_m | 0.03113214 | 2.910796e+00 | 0.9915 |
| 1. frequency_in_a_month | accessibility_distance_m:education_levelsecondary | -0.03076928 | 2.910797e+00 | 0.9916 |
| 1. frequency_in_a_month | accessibility_distance_m | -0.02346327 | 4.929922e-03 | 0.0000 *** |
| 1. as.numeric(mediator_motivation) | perception_in_relation_to_healthgood | -7.36011278 | 2.845713e+00 | 0.0112 * |
| 1. health response | frequency_in_a_month | 0.12568750 | 7.777633e-02 | 0.1093 |
| 1. health response | as.numeric(mediator_motivation) | 0.03619454 | 2.520970e-02 | 0.1511 |
| 1. health response | education_levelsecondary | -82.96730892 | 7.239419e+03 | 0.9909 |
| 1. health response | education_leveltertiary | -80.18112211 | 7.239419e+03 | 0.9912 |
| 1. health response | education_levelsecondary:accessibility_distance_m | 0.06771083 | 6.786937e+00 | 0.9920 |
| 1. health response | accessibility_distance_m | -0.06575755 | 6.786937e+00 | 0.9923 |
| 1. health response | frequency_in_a_month | -1.10226541 | 1.150328e+02 | 0.9924 |
| 1. health response | education_leveltertiary: accessibility_distance_m | 0.06381840 | 6.786937e+00 | 0.9925 |
| 1. health response | frequency_in_a_month:education_leveltertiary | 1.06595666 | 1.150328e+02 | 0.9926 |
| 1. health response | frequency_in_a_month:education_levelsecondary | 1.04839889 | 1.150328e+02 | 0.9927 |
