# Supplementary material for: Effects of urban green spaces on human perceived health improvements: Provision of green spaces is not enough but how people use them matters
Source: PLoS One. 2020 Sep 23;15(9):e0239314. doi: 10.1371/journal.pone.0239314 (PMC7510974; doi:10.1371/journal.pone.0239314)
Supplement: S12 Table — See R scripts in S2 File for details of the meta-model. * indicates significant relationships between predictor and response. (DOC) [file pone.0239314.s014.doc]

**S12 Table. Path coefficients of meta-model 11 defined in Figure 2. See R scripts in SI-4 for details of the meta-model. * indicates significant relationships between predictor and response.**

| **response** | **predictor** | **estimate** | **Std.error** | **p.value** |
| --- | --- | --- | --- | --- |
| 1. perception_in_relation_to_health | education_leveltertiary | 33.370395896 | 2.910796e+03 | 0.9909 |
| 1. perception_in_relation_to_health | education_levelsecondary | 32.392518752 | 2.910796e+03 | 0.9911 |
| 1. perception_in_relation_to_health | accessibility_distance_m:education_leveltertiary | -0.032075525 | 2.910797e+00 | 0.9912 |
| 1. perception_in_relation_to_health | accessibility_distance_m | 0.031132137 | 2.910796e+00 | 0.9915 |
| 1. duration_hour | accessibility_distance_m:education_levelsecondary | 0.031132137 | 2.910796e+00 | 0.9915 |
| 1. duration_hour | accessibility_distance_m | -0.030769284 | 2.910797e+00 | 0.9916 |
| 1. as.numeric(mediator_motivation) | perception_in_relation_to_healthgood | 0.001422771 | 4.848527e-04 | 0.0042 ** |
| 1. health response | duration_hour | 0.365119808 | 2.798729e-01 | 0.1952 |
| 1. health response | as.numeric(mediator_motivation) | -1.330600487 | 8.224546e-01 | 0.2905 |
| 1. health response | education_levelsecondary: accessibility_distance_m | 0.025307491 | 2.394032e-02 | 0.9919 |
| 1. health response | accessibility_distance_m | 0.068611752 | 6.786938e+00 | 0.9923 |
| 1. health response | duration_hour | -0.065909969 | 6.786937e+00 | 0.9923 |
| 1. health response | duration_hour:education_leveltertiary | 32.701909545 | 3.393469e+03 | 0.9923 |
| 1. health response | duration_hour:education_levelsecondary | -32.624058200 | 3.393469e+03 | 0.9924 |
| 1. health response | education_leveltertiary: accessibility_distance_m | -32.529276264 | 3.393469e+03 | 0.9924 |
| 1. health response | education_levelsecondary | 0.064823334 | 6.786938e+00 | 0.9972 |
| 1. health response | education_leveltertiary | -18.651398937 | 5.365545e+03 | 0.9977 |
