# Supplementary material for: Effects of urban green spaces on human perceived health improvements: Provision of green spaces is not enough but how people use them matters
Source: PLoS One. 2020 Sep 23;15(9):e0239314. doi: 10.1371/journal.pone.0239314 (PMC7510974; doi:10.1371/journal.pone.0239314)
Supplement: S13 Table — See R scripts in S2 File for details of the meta-model. * indicates significant relationships between predictor and response. (DOC) [file pone.0239314.s015.doc]

**S13 Table. Path coefficients of meta-model 12 defined in Figure 2. See R scripts in SI-4 for details of the meta-model. * indicates significant relationships between predictor and response.**

| **response** | **predictor** | **estimate** | **Std.error** | **p.value** |
| --- | --- | --- | --- | --- |
| 1. perception_in_relation_to_health | education_leveltertiary | 33.3703958958 | 2.910796e+03 | 0.9909 |
| 1. perception_in_relation_to_health | education_levelsecondary | 32.3925187519 | 2.910796e+03 | 0.9911 |
| 1. perception_in_relation_to_health | accessibility_distance_m:education_leveltertiary | -0.0320755252 | 2.910797e+00 | 0.9912 |
| 1. perception_in_relation_to_health | accessibility_distance_m | 0.031132136 | 2.910796e+00 | 0.9915 |
| 1. perception_in_relation_to_health | accessibility_distance_m:education_levelsecondary | -0.0307692844 | 2.910797e+00 | 0.9916 |
| 1. intensity | perception_in_relation_to_healthgood | 1.3722196595 | 7.858704e-01 | 0.0808 |
| 1. intensity | accessibility_distance_m | 0.0001770277 | 1.002454e-03 | 0.8598 |
| 1. duration_hour | intensity | 7.8614916286 | 2.034683e+00 | 0.0002*** |
| 1. as.numeric(mediator_motivation) | education_levelsecondary:accessibility_distance_m | 0.0011532556 | 2.345820e-03 | 0.6230 |
| 1. health response | education_levelsecondary | -1.1301960797 | 2.463163e+00 | 0.6463 |
| 1. health response | accessibility_distance_m | 0.0004671798 | 1.547863e-03 | 0.7628 |
| 1. health response | as.numeric(mediator_motivation) | -0.0087923004 | 2.918230e-02 | 0.7632 |
| 1. health response | education_leveltertiary | -0.2036752690 | 1.511607e+00 | 0.8928 |
| 1. health response | intensity | 18.7864699730 | 6.522639e+03 | 0.9977 |
| 1. health response | intensity:education_leveltertiary | 6.522639e+03 | 6.787762e+03 | 1.0000 |
| 1. health response | intensity:education_levelsecondary | -0.0537482234 | 6.746457e+03 | 1.0000 |
